# A QTL analysis of host plant effects on fungal endophyte biomass and alkaloid expression in perennial ryegrass

**DOI:** 10.1007/s11032-015-0350-1

**Published:** 2015-07-18

**Authors:** Marty J. Faville, Lyn Briggs, Mingshu Cao, Albert Koulman, M. Z. Zulfi Jahufer, John Koolaard, David E. Hume

**Affiliations:** Grasslands Research Centre, AgResearch Ltd., PB 11008, Palmerston North, New Zealand; Ruakura Research Centre, AgResearch Ltd., PB 3123, Hamilton, New Zealand; Elsie Widdowson Laboratory, MRC Human Nutrition Research, 120 Fulbourn Road, Cambridge, CB1 9NL UK

**Keywords:** DIMSMS, Endophyte, ELISA, *Epichloë*, *Lolium*, Metabolite profiling, QTL

## Abstract

The association between perennial ryegrass (*Loliumperenne* L.) and its *Epichloë* fungal endophyte symbiont, *Epichloëfestucae* var. *lolii*, supports the persistence of ryegrass-based pastures principally by producing bioactive alkaloid compounds that deter invertebrate herbivory. The host plant genotype affects endophyte trait expression, and elucidation of the underlying genetic mechanisms would enhance understanding of the symbiosis and support improvement of *inplanta* endophyte performance through plant breeding. Rapid metabolite profiling and enzyme-linked immunosorbent assay were used to quantify endophyte alkaloids and mycelial mass (MM) in leaves harvested, in consecutive autumns, from an *F*_1_ mapping population hosting standard toxic endophyte. Co-aligned quantitative trait loci (QTL) on linkage groups (LG)2, LG4 and LG7 for MM and concentrations of alkaloids peramine and ergovaline confirmed host plant effects on both MM and alkaloid level and inferred the effect on alkaloids was modulated through the quantity of endophyte present in the leaf tissue. For ergovaline, host regulation independent of endophyte concentration was also indicated, by the presence of MM-independent ergovaline QTL on LG4 and LG7. Partitioning of host genetic influence between MM-dependent and MM-independent mechanisms was also observed for the alkaloid *N*-formylloline (NFL), in a second mapping population harbouring a tall fescue-sourced endophyte. Single-marker analysis on repeated MM and NFL measures identified marker–trait associations at nine genome locations, four affecting both NFL and MM but five influencing NFL concentration alone. Co-occurrence of QTL on LG3, LG4 and LG7 in both mapping populations is evidence for host regulatory loci effective across genetic backgrounds and independent of endophyte variant. Variation at these loci may be exploited using marker-assisted breeding to improve endophyte trait expression in different host population × endophyte combinations.

**Electronic supplementary material:**

The online version of this article (doi:10.1007/s11032-015-0350-1) contains supplementary material, which is available to authorized users.

## Introduction

Perennial ryegrass (*Loliumperenne* L.) is a major source of nutrition for livestock grazing permanent pastures in temperate, mesic regions of the world, including New Zealand (Jung et al. 1996). The persistence of a large portion of New Zealand perennial ryegrass pasture is sustained by a symbiotic association (Thom et al. 2012a; Young et al. 2013) between perennial ryegrass and the naturally infecting endophytic *Epichloë* fungal species *Epichloëfestucae* var. *lolii* (Leuchtmann et al. 2014), previously classified as *Neotyphodiumlolii*.

Bioactive alkaloids produced by the endophyte *inplanta* confer benefits to the grass host, principally through the deterrence of herbivory by invertebrate pests. Key alkaloids produced *inplanta* by the prevalent, New Zealand standard toxic *E*. *festucae* var. *lolii* endophyte (van Zijll de Jong et al. 2008) include the pyrrolopyrazine alkaloid, peramine; the ergopeptine alkaloid, ergovaline; and the indole-diterpene, lolitrem B. Each of these alkaloids protects the host plant against insect damage. Peramine is a causal factor in the deterrence of feeding by Argentine stem weevil (*Listronotusbonariensis* Kuschel) (Rowan and Gaynor 1986), a significant pest in New Zealand pastures (Prestidge and Ball 1995). Ergovaline inhibits herbivory by Argentine stem weevil (Prestidge and Ball 1995) and contributes to resistance against African black beetle (*Heteronychusarator* F.) (Ball et al. 1997), a destructive pasture pest which is not affected by peramine. Lolitrem B also offers protection against Argentine stem weevil (Prestidge and Ball 1995).

Ergovaline and lolitrem B are also causative agents in mammalian mycotoxicoses (Fletcher and Easton 1997) which, in agriculture, is an undesirable feature of the symbiosis that negatively affects animal performance (Young et al. 2013). This has been addressed by discovering and then artificially infecting perennial ryegrass cultivars with selected non-toxic strains of *E*. *festucae* var. *lolii* (Johnson et al. 2013) to generate novel ryegrass–endophyte associations, such as the AR1 strain widely used in New Zealand agriculture. AR1 produces an alkaloid phenotype (peramine, no ergovaline or lolitrem B) deterrent to insect pests while non-toxic to grazing livestock (Johnson et al. 2013). Competence against a broad spectrum of invertebrate pests, including African black beetle, may also be achieved via ryegrass–endophyte associations that continue to produce alkaloids toxic to mammals, such as ergovaline. In this scenario, levels of ergovaline need to be low enough to minimise negative mammalian health effects, while high enough to preserve bioactivity against insects (Easton et al. 2002; Hill et al. 2002; Thom et al. 2012a).

A more recent extension of the strategy of developing novel ryegrass–endophyte associations has seen ryegrasses inoculated with *Epichloë* symbionts normally found in natural association with other grass species (Easton 2007), including *E*. *uncinata* (hosted by meadow fescue, *Festucapratensis* L.), *E*. *coenophiala* and *Epichloë* sp. FaTG-3 (both hosted by tall fescue, *F*. *arundinacea* Schreb.). These endophytes, when in their natural hosts, produce high concentrations of loline (aminopyrrolizidine) alkaloids which are of particular value in grass-based forage systems: lolines are a class of secondary metabolites (Schardl et al. 2007) that are non-toxic to mammals (Bush et al. 1997) while exhibiting potent, broad spectrum deterrence to a range of insect pests (Popay and Bonos 2005) and are not produced in natural ryegrass–endophyte associations. Loline alkaloid composition varies amongst different tall fescue and meadow fescue endophyte associations, but, with exceptions, the major loline alkaloid produced is *N*-formylloline, others being *N*-acetylloline and *N*-acetylnorloline (Ball et al. 2006).

With each of the described approaches to qualitative modification of endophyte alkaloid profiles in ryegrass, regulation of alkaloid quantity (higher concentrations for favourable alkaloids, downward regulation of those with mammalian toxicity) may improve pasture performance from these ryegrass–endophyte associations. The specific genetic background of a host plant has a significant influence on variation in endophyte alkaloid levels in grass herbage, and heritability for these traits has been reported as moderate to high (*h*^2^ 0.49–0.72) (Adcock et al. 1997; Easton et al. 2002; Easton 2007). Therefore, manipulation of *inplanta* endophyte alkaloid levels may be realised by recurrent selection of host plants that are genetically optimal for endophyte trait expression (Adcock et al. 1997). Marker-assisted selection (MAS) approaches, based on linked markers or functional gene variants, may enhance the efficiency of selection compared with direct selection alone. MAS to exploit host plant modulation of symbiont trait expression has been explored in other plant species, from the perspectives of mitigating fungal pathogenicity (Paul et al. 2003; Brooks et al. 2005; Horsley et al. 2006) or enhancing arbuscular mycorrhizal (Barker et al. 2002; Galván et al. 2011) and rhizobial symbioses (Nodari et al. 1993; Souza et al. 2000; Ramaekers et al. 2013). In the ryegrass–endophyte context, MAS would enable precise, early and efficient host plant selection, reducing the time and high costs associated with inoculating large numbers of selection candidates with endophyte and subsequently measuring endophyte traits *inplanta* prior to selection.

Our objective was to identify perennial ryegrass quantitative trait loci (QTL) that influence *inplanta* levels of endophyte mycelial mass (MM) and the alkaloids peramine (PER), ergovaline (EGV) and *N*-formylloline (NFL), in contrasting *F*_1_ perennial ryegrass mapping populations containing different endophytic *Epichloë* species. To facilitate analysis of the large sample numbers needed for QTL analysis, rapid metabolite profiling methodology (Koulman et al. 2009) was employed and a modified enzyme-linked immunosorbent assay (ELISA) for the quantification of MM *inplanta* was developed and is described.

## Materials and methods

### Mapping populations and phenotyping trials

An *F*_1_ mapping population (*I* × *S*) from a pair cross between two diploid heterozygous genotypes from a long-rotation hybrid ryegrass (*L*. *boucheanum* syn. *L*. *hybridum*) cultivar ‘Grasslands Impact’ (parent ‘I’) and a perennial ryegrass cultivar ‘Grasslands Samson’ (parent ‘S’) (Sartie et al. 2011; Faville et al. 2012) was used for QTL discovery for PER, EGV and MM. Parent ‘I’ was infected with a naturally occurring standard toxic endophyte (*Epichloëfestucae* var. *lolii)* strain (SE) which produces both PER and EGV, as well as lolitrem B, *inplanta*. *I* × *SF*_1_ mapping population progeny were maternally derived from parent ‘I’ only and therefore also contained SE, due to the obligate vertical maternal transmission of endophyte. Three clonal replicates of 190 genotypes (parents plus 188 *F*_1_ progeny) were grown outdoors in 1.5-L pots filled with a commercial sand-peat (40: 60) potting mixture, at AgResearch Grasslands Research Centre, Palmerston North, New Zealand (40°21′S, 175°37′E), in a randomised complete block experimental design. Plants were cut back to 4 cm above the soil surface, 4 weeks (2005) or 2 weeks (2006) prior to harvesting material for metabolite and MM analyses. The regrowth period in 2006 was reduced in an effort to promote higher EGV expression in leaf tissue. Bulk herbage samples (leaf lamina and a small amount of sheath) were harvested, in the Southern Hemisphere mid-autumn, to 4 cm above the soil surface, transferred directly into liquid nitrogen and subsequently stored at −20 °C. Samples were later freeze-dried and milled through a 1-mm sieve using a UDY Cyclone Sample Mill (UDY Corporation, Fort Collins, CO) in preparation for phenotypic analyses (metabolite profiling and ELISA). Samples were from two replicates per genotype on 31 March 2005 and from three replicates per genotype on 3 April 2006. An improved extraction process for metabolite profiling enabled more samples to be processed in 2006 than in 2005.

A second *F*_1_ biparental population (*P* × *O*), originating from a pair cross between single heterozygous genotypes from tetraploid ryegrass breeding population ‘2001’ and tetraploid long-rotation hybrid ryegrass cultivar ‘Grasslands Ohau’, respectively, was used for the analysis of NFL and MM. ‘2001’ is a selection originating from a cross amongst tetraploid Festulolium cultivars of the *Festucapratensis* x *Loliummultiflorum* type (Dr Alan Stewart pers comm). *P* × *OF*_1_ progeny were derived from seed harvested exclusively from the ‘2001’ parent (parent ‘P’), which was artificially infected with a non-ergovaline producing endophyte strain (*E*. *sp*. FaTG-3) from tall fescue, designated AR501. In contrast to alkaloid expression in its natural host, in perennial ryegrass AR501 produces a loline profile consisting only of NFL and no other recognised loline alkaloid (Ball et al. 2006). Three clonal replicates of 285 *F*_1_ progeny genotypes were grown outdoors in 1.5-L pots filled with a commercial sand-peat potting mixture, at AgResearch Grasslands, Palmerston North, New Zealand, beginning March 2010. Each genotype was replicated three times in a repeated row-column design which was optimised to avoid clonal replicates being present more than once in the same row or column.

Plants were cut back to 4 cm above the soil surface, 3.5–4.5 weeks (variation in timing enforced by variation in growth rates at different times of the year) prior to harvesting herbage for NFL and MM analyses. Leaf lamina were harvested directly above the pseudostem, from all three replicates per genotype, into liquid nitrogen on 1 April 2010, 18 May 2010, 16 February 2011, 2 May 2011 and 29 September 2011 and stored at −20 °C. Samples were then freeze-dried and milled, as described for population *I* × *S*. Plants in this trial were split and repotted as even-sized ramets between years, in December 2010.

### ELISA analysis of endophyte mycelial mass

#### Herbage extraction

Twenty milligram (±2 mg) of the freeze-dried and milled material was weighed into a glass Kimax tube (15 mL) and extracted with 0.05 % Tween 20 in phosphate-buffered saline (phosphate-buffered saline in Tween, PBST, 10 mL) for 3 h at 30 °C. The extracts were centrifuged (Eppendorf Microfuge 5415 C) at 5400*g* for 3 min, and an aliquot of the supernatant was analysed, undiluted and also diluted in PBST, for MM by the competitive ELISA described below. Extract supernatants were stored overnight at 4 °C before analysis or for longer-term storage at −20 °C. Independent extractions were made from two (2005) or three (2006) replicates of all 188 *I* × *S* population genotypes and all three replicates of 285 *P* × *O* population genotypes sampled in May 2011 and September 2011.

#### Competitive ELISA

The presence of endophyte mycelia in sample extracts was indicated by inhibition of specific antibody binding to coating antigen. All assay procedures were carried out at 21 °C. Freeze-dried coating antigen previously prepared from *E*. *festucae* var. *lolii* mycelium following the method of Ball et al. (1995) was diluted (20 µg/mL) in standard ELISA coating buffer, carbonate/bicarbonate buffer (50 mM, pH 9.6), and microtiter plates coated (100 µL/well). After incubation for 16 h, plates were washed four times with PBST and blocked for 1 h with 1 % BSA (bovine serum albumin) in PBST (200 µL/well). This was followed by four washes with PBST.

Standards and samples were preincubated with the anti-endophyte antibody. ELISA standard curves were prepared for each assay using *E*. *festucae* var. *lolii* endophyte prepared by Ball et al. (1995) as the reference standard. Standard was diluted in PBST to 200 µg/mL, and further 2.5-fold serial dilutions were made in PBST to give 10 standards (0.05–200 µg/mL). To each tube, 150 µL of standard or sample extract was added, followed by 150 µL of diluted anti-*E*. *festucae* var. *lolii* antibodies (Ball et al. 1995). Antibodies were diluted in 1 % BSA in PBST such that the maximum absorbance (*A*_max_) in the assay, in the absence of analyte, was approximately 1.0 absorbance. After tubes were incubated for 2 h, 100 µL of the standard or sample extract and antibody mixture were added to each well on blocked plates. All standards and samples were analysed in duplicate wells. Variation within each plate (intra-assay) was determined by preparing duplicate extracts for two of the samples analysed on each plate. Between-plate variation (inter-assay) was determined by analysing the same positive control sample on each plate.

Plates were incubated for 1 h, and after four washes (PBST), 100 µL of goat anti-rabbit-HRP (horseradish peroxidise; Dako, Australia) diluted 1:10,000 in 1 % BSA in PBST was added. Plates were incubated for 2 h and washed four times with PBST. K-Blue Aqueous TMB (3,3′,5,5′ tetramethylbenzidine) substrate (Neogen Corporation, USA) for HRP was added (100 µL/well), and after incubation with shaking for 30 min, the enzyme reaction was stopped by addition of sulphuric acid (0.3 M, 100 µL/well).

The absorbance of wells was determined at 450 nm using a Versamax microplate reader (Molecular Devices Corporation, USA). Data analysis was performed using SOFTmax PRO data analysis software (Molecular Devices Corporation). Curve fits of mean absorbance versus the logarithm of the analyte concentration were performed by four-parameter curve fit. Results were reported as *E*. *festucae* var. *lolii* immunoreactive equivalents (IRE) in mg/g DW.

#### ELISA development and validation

Perennial ryegrass samples that had been previously analysed by immunoblot (Simpson et al. 2012) and shown to be free of endophyte were milled, and the effects of dilution of endophyte-free grass extract on ELISA *A*_max_ were studied for each sample extracting system investigated. The system selected was the one requiring the least dilution of grass extracts to remove inhibition of *A*_max_ and giving the best assay signal (inhibition of colour development) with endophyte-containing samples extracted in the same system and at the same dilution. Minimum sample size giving maximum extraction of immunoreactivity was also determined (data not presented).

Within-plate variation was determined by duplicate extraction and analysis of six replicates of a single sample on the same plate. Between-plate variation was determined by extraction and analysis of a sample on 12 different occasions.

### Metabolite profiling of PER and EGV in population *I* × *S*

Analysis of *I* × *S* ryegrass herbage samples for endophyte alkaloid secondary metabolites PER *(m/z* 248) and EGV (*m/z* 534) was completed using direct infusion mass spectrometry metabolite profiling procedures (DIMSMS) as described in Koulman et al. (2009). The polar isopropanol–water (1:1) extraction solvent used extracts PER and EVG efficiently but does not extract lolitrem B (Cao et al. 2008); therefore, the latter alkaloid was not assessed in this study. Data from 2005 were generated using an untargeted metabolite profiling DIMSMS method described by Koulman et al. (2007). The accuracy of DIMSMS estimation of peramine quantity was validated by LC–MS on a subset of samples and for ergovaline by HPLC with fluorescence detection (Koulman et al. 2009). Due to low throughput of the DIMSMS method, no more than two clonal replicates per genotype were analysed from the 2005 data set. Data for 2006 were generated using a targeted DIMS(MS) approach that more than doubles the throughput for specific compounds (Koulman et al. 2009). This was more resource efficient and enabled all three clonal replicates to be analysed. Data were reported as normalised intensity units (NIU).

### Quantitation of NFL in population *P* × *O*

Analysis of 4275 *P* × *O* herbage samples for NFL (five time points x three replicates × 285 *F*_1_ progeny genotypes) was conducted using a new extraction methodology and competitive ELISA (L Briggs et al., unpublished data) to be described in a future publication. Briefly, freeze-dried and milled herbage was extracted with PBS for 1 h, microtitre plates were coated with hemisuccinyl loline–ovalbumin coating conjugate, and BSA was used as a blocking agent. ELISA standards were prepared with NFL as the reference standard (working range 0.1–400 ng/mL), and results were reported in NFL IRE µg/g DW. After 300- and 600-fold dilution of extracts, the limit of quantitation for NFL was 3 µg/g in dried herbage.

### Statistical analysis of phenotypic data

Alkaloid and MM data from the population *I* × *S* and *P* × *O* data sets were analysed using the variance component analysis procedure, residual maximum likelihood (REML) option, in GenStat (2006). A completely random linear model was used in the analysis using the REML algorithm. The final, adjusted genotypic means were based on best linear unbiased predictors (BLUPs). Estimation of the significance of the genotypic variance component for each trait was based on the log-likelihood ratio test. The linear models also included genotype-by-year and genotype-by-month interaction effects in the analysis of the *I* × *S* and *P* × *O* data sets, respectively. The variance components generated from the REML analysis were used to estimate clonal mean repeatability (*R*_c_,) the upper limit of the degree of genetic determination (Falconer 1989), for the traits PER, EGV, NFL and MM. The model $R^{1}_{\text{c}} = \, \sigma^{2}_{\text{g}} /\left( {\sigma^{2}_{\text{g}} + \sigma^{2}_{\varepsilon } /n_{\text{r}} } \right)$ was used for calculating clonal mean repeatability using individual year (*I* × *S*) or month (*P* × *O*) trait data, where $\sigma^{2}_{\text{g}}$ is the genotypic component of variance, $\sigma^{2}_{\varepsilon }$ the residual variance and *n*_r_ the number of replications. The model $R^{2}_{\text{c}} = \, \sigma^{2}_{\text{g}} /\left( {\sigma^{2}_{\text{g}} + \, \sigma^{2}_{\text{gy}} /n_{\text{y}} + \sigma^{2}_{\varepsilon } /n_{\text{r}} } \right)$ was based on variance components generated from the genotype-by-year analysis in *I* × *S*, where $\sigma^{2}_{\text{gy}}$ is the genotype-by-year component of variance and *n*_y_ is the number of years. Similarly, the model $R^{3}_{\text{c}} = \, \sigma^{2}_{\text{g}} /\left( {\sigma^{2}_{\text{g}} + \, \sigma^{2}_{\text{gm}} /n_{\text{m}} + \sigma^{2}_{\varepsilon } /n_{\text{r}} } \right)$ was based on variance components from the genotype-by-month analysis in *P* × *O*, where $\sigma^{2}_{\text{gm}}$ is the genotype-by-month component of variance and *n*_m_ the number of months. Pearson correlations amongst traits were obtained using GenStat (2012).

### Multiple QTL mapping analysis in population *I* × *S*

An existing integrated genetic linkage map of the *I* × *S* population, based on EST-derived simple sequence repeat (SSR) and sequence-tagged site (STS) markers (Sartie et al. 2011), was used for QTL analysis. This map was augmented for the current study by a further 30 ryegrass and tall fescue SSR markers (Khaembah et al. 2013).

QTL analysis was performed with MapQTL^®^ 4.0 software (Van Ooijen et al. 2002) under the CP population mode, using 2005 and 2006 BLUPs for MM, PER and EGV from *n* = 188 *F*_1_ progeny. Additionally, QTL analysis was conducted using BLUPs, generated from a genotype-by-year analysis, integrating values for traits MM and PER across both years (designated MM_int_ and PER_int_, respectively). Interval mapping was performed first, and then the estimated positions and magnitude of QTL were refined using the multiple QTL mapping (MQM) module, as described in Sartie et al. (2011). Permutation testing (*n* = 2000) was performed for each measurement to establish logarithm-of-odds (LOD) thresholds for QTL declaration at a linkage group- or genome-wide significance of *P* ≤ 0.05 (Churchill and Doerge 1994). QTL position was described by LOD peak position and 2-LOD support intervals. An additional criterion for declaration of a significant QTL was the presence of markers within the 2-LOD support interval that were significant by Kruskal–Wallis analysis, as executed in MapQTL^®^ 4.0. QTL naming convention is q-trait-year-linkage group, where trait is MM, PER or EGV, year is 05 (2005) or 06 (2006), and linkage group (LG) is between 1 and 7, corresponding to ryegrass LG1–LG7.

Phenotype means for the four different expected QTL genotype classes (ac, ad, bc and bd) were calculated in MapQTL^®^ 4.0. These data were used to report QTL in terms of the individual parental effects (i.e. the difference in effect of the alleles inherited from each parent, ‘I’ and ‘S’), following the model of Knott et al. (1997), as used by Sewell et al. (2000). At each QTL, individual effects (difference in effect of the alleles inherited from each parent) for the maternal (parent ‘*I*’) and paternal (parent ‘*S*’) parents were estimated as described by Sartie et al. (2011).

### Single-marker analysis in population *P* × *O*

For population *P* × *O*, parental genotypes were not available for genotyping, preventing genetic linkage analysis and genetic map-based QTL analysis. Marker–trait associations were consequently identified based on single-marker tests conducted using the nonparametric Kruskal–Wallis one-way analysis of variance (ANOVA), implemented in MapQTL^®^ 4.0. This was used, rather than parametric ANOVA, because of unbalanced SSR genotype proportions in the mapping population (refer to Table 4).

A total of 153 SSR markers, distributed throughout the *I* × *S* genetic linkage map, were used to genotype 180 randomly selected *P* × *OF*_1_ progeny. Plants were sampled for DNA isolation by excising 100–200 mg basal tiller tissue. Total genomic DNA was isolated from tiller tissue using the FastDNA kit (MP Biomedicals, Solon, OH, USA) following the manufacturer’s instructions for fresh plant tissue. DNA extracts were diluted by a factor of 10 prior to SSR assay. PCR amplifications were completed in a 10-μL reaction volume as described in Faville et al. (2004), except that a final concentration of 2.5 mM magnesium chloride and 0.75 units of Platinum Taq DNA polymerase (Life Technologies, Carlsbad, California, USA) were used. PCR was performed on iCycler thermocyclers (BioRad, Hercules, California, USA). Capillary electrophoresis and subsequent genotype calling were performed as described by Sartie et al. (2011).

Marker alleles were scored as individual dominant markers (1 = present, 0 = absent), and those that segregated within a frequency interval of 0.20–0.80 in the *F*_1_ progeny were identified and selected for further use. Markers meeting this criterion were used for Kruskal–Wallis analysis of MM and NFL BLUPs generated from each individual sampling time as well as for BLUPs from a genotype-by-month analysis integrating all sampling time points (designated MM_int_ and NFL_int_, respectively). For Kruskal–Wallis tests, a marker allele–trait association was accepted when at least one trait measurement was significant at that locus, at *P* < 0.005. When this criterion was met, tests for other individual trait measurements at this locus that were significant at the lower threshold of *P* < 0.01 were also retained.

## Results

### MM ELISA development

Endophyte preparations were investigated for suitability as plate coaters used in combination with anti-*E*. *festucae* var. *lolii* antibodies. An ELISA with acceptable performance characteristics was established, and extracts from endophyte-free and endophyte-containing herbage samples were investigated using PBS containing 0.5 % Tween 20, PBST and PBST containing 10 % methanol. The extractant selected was PBST as this provided best discrimination between positive and negative extracts. Minimum sample size giving maximum extraction of *E*. *festucae* var. *lolii* IRE was found to be 20 mg. The limit of quantitation (IC_20_) for *E*. *festucae* var. *lolii* IRE in pasture samples, as determined by the ELISA, was 0.9 mg/g in dried herbage. Using the optimised assay, within-assay coefficient of variation for six replicates was 5.0 %, while mean coefficient of variation for between assay variation determined on 12 separate occasions was 1.7 %.

### Phenotypic evaluation of MM and alkaloids in population *I* × *S*

Data distributions for endophyte traits in population *I* × *S* are shown in Fig. S1. In general, BLUP distributions were normal with some skewedness towards lower values for MM 2005 and EGV 2005 and 2006 (Fig. S1, Table 1). Statistically significant (*P* < 0.01) genotypic variation ($\sigma^{2}_{\text{g}}$) was indicated for all traits in each year except EGV 2005 and, furthermore, significant genotypic variation was determined for both MM and PER across the two years (Table 1). Significant (*P* < 0.05) genotype-by-year interactions ($\sigma^{2}_{\text{gy}}$) were identified for both MM and PER but not for EGV (Table 1). For MM, the ratio of $\sigma^{2}_{\text{g}} /\sigma^{2}_{\text{gy}}$ was 3.05, in contrast to PER for which the same ratio was 0.52, indicating a considerably stronger interaction effect on PER. On average, MM, EGV and PER values were higher amongst *I* × *SF*_1_ progeny in 2006 compared with 2005 (Table 1). The progeny clone mean repeatability, *R*_c_, represents the proportion of total phenotypic variance due to genotypic variation, the upper limit to the degree of genetic determination for the trait (Falconer 1989). *R*_c_ values (Table 1) were moderate for MM 2005 and all three traits from the 2006 data set (0.56–0.66), but *R*_c_ for PER 2005, at 0.22, was considerably lower, while it was not calculated for EGV 2005 due to no significant genotypic variance. Very weak to moderate phenotypic correlation amongst traits was indicated by significant (*P* < 0.01) positive correlation coefficients ranging from 0.20 to 0.64 (Table S1). Correlations amongst traits were highest in the 2006 data set, and within that data set PER was more highly correlated with MM than was EGV (Table S1). Correlations between equivalent 2005 and 2006 measurements were very weak for PER and moderate for MM. The low *R*_c_ for PER 2005 and the weaker correlations associated with the 2005 traits were likely influenced by the lower number of clonal replicates as well as a relatively high proportion of missing data in the data sets used to estimate the alkaloid BLUP adjusted means (16 % in 2005 compared with 2 % in 2006).

**Table 1:** Mean trait values, ranges, least significant differences (LSD), genotypic variance component ($\sigma^{2}_{\text{g}}$) and genotype-by-year interaction variance component ($\sigma^{2}_{\text{gy}}$), with associated standard errors (SE), and progeny clone mean repeatability (*R*_c_), for endophyte-related traits measured in 200 perennial ryegrass *I* × *S* *F*_1_ mapping population progeny and two parental genotypes (Parents ‘*I*’ and ‘*S*’), grown in pots outdoors at Palmerston North, New Zealand, during 2005–2006

Trait	Year	*F*_1_ progeny mean^a^	*F*_1_ progeny range^a^	Parent ‘*I*’ mean^a^	Parent ‘*S*’ mean^a^	LSD (*P* < 0.05)	$\sigma^{2}_{\text{g}}$ ± SE^b^	$\sigma^{2}_{\text{gy}}$ ± SE^b^	*R*_c_
MM	2005	4.7	2.1–17.7	6.3	2	1.37	1.44 ± 0.22		0.66
MM	2006	9.3	3.8–18.5	10.8	3	2.48	4.20 ± 0.69		0.63
MM	Integrated	7	4.1–12.3	8.4	3.1	1.71	2.07 ± 0.69	0.68 ± 0.31	0.55
PER	2005	17.8	5.5–30.4	18.3	13.1	3.69	4.70 ± 2.27		0.22
PER	2006	26.9	11.6–43.9	24.7	9	6.64	24.69 ± 4.67		0.52
PER	Integrated	23	18.3–28.5	21.9	12.1	4.46	5.46 ± 2.56	10.43 ± 3.34	0.26
EGV	2005	5.8	2.8–11.1	6.5	7.4	0.65	0.09 ± 0.23		–
EGV	2006	8.3	1.7–21.3	7.1	2	3.57	7.60 ± 1.37		0.56

Data are presented for endophyte mycelial biomass ELISA (MM, in immunoreactive equivalents, IRE mg/g DW), peramine (PER, in normalised intensity units) and ergovaline (EGV, normalised intensity units) measured in bulk herbage harvested in March 2005 (*n* = 2 replicates) and April 2006 (*n* = 3 replicates) and also calculated across both years (Integrated). *R*_c_ for EGV 2005 was not calculated due to no significant genotypic variance, and genotype-by-year analysis for EGV was also not completed for that reason. Parent ‘*S*’ contains endophyte AR6, while NZ standard toxic endophyte is present in all other mapping population genotypes

^a^Data are expressed as immunoreactive equivalent units (IRE) mg/g DW for MM and normalised intensity units (NIU) for PER and EGV

^b^All significant at *P* < 0.01, except EGV 2005

### Phenotypic evaluation of MM and NFL in population *P* × *O*

In population *P* × *O*, significant skewedness was observed for all traits (data not shown), except MM May 2011 and NFL May 2010, and data for the affected traits were consequently square root-transformed (Fig. S2) prior to Kruskal–Wallis analysis. Statistically significant (*P* < 0.01) genotypic variation was observed for all traits at each monthly time point as well as across all months (Table 2). Mean NFL amongst *P* × *OF*_1_ progeny varied between measurement time points (Table 2). NFL was higher in autumn months (April 2010 and May 2011) in both years (Table 2) and lowest in summer (February 2011) and spring (September 2011) and, similarly, the population mean for MM was higher in May 2011 than September in the same year (Table 2). *R*_c_ values estimated for NFL and MM were moderate to high (Table 2), ranging from 0.65 (NFL February 2011) to 0.93 (MM May 2011). Pairwise phenotypic correlations (*P* < 0.05) amongst the five NFL measurements were moderate (mean *r* = 0.56) and stronger between the two MM measurements (*r* = 0.77) (Table S1), and significant (*P* < 0.05) genotype-by-month interaction ($\sigma^{2}_{\text{gm}}$) was confirmed for both traits (Table 2). For MM, the ratio of $\sigma^{2}_{\text{g}} /\sigma^{2}_{\text{gy}}$ was 1.96, almost twice that of NFL (1.05), indicating greater influence of the interaction component on NFL. Weak-to-moderate, significant phenotypic correlations between NFL and MM were determined at both the May and September 2011 time points (mean *r* = 0.41).

**Table 2:** Mean trait values (BLUPs), ranges, least significant differences (LSD), genotypic variance component ($\sigma^{2}_{\text{g}}$) and genotype-by-month interaction variance component ($\sigma^{2}_{\text{gm}}$) with associated standard errors (SE) and progeny clone repeatability (*R*_c_), for endophyte traits measured in 285 perennial ryegrass *P* × *O* *F*_1_ mapping population progeny infected with endophyte AR501

Trait	Month	Year	*F*_1_ progeny mean^a^	*F*_1_ progeny range^a^	LSD (*P* < 0.05)	$\sigma^{2}_{\text{g}}$ ± SE^b^	$\sigma^{2}_{\text{gm}}$ ± SE^b^	*R*_c_
NFL	April	2010	97.3	10.3–313.4	2.07	5.96 ± 0.62		0.82
NFL	May	2010	61.2	14.5–119.7	19.93	407.0 ± 45.8		0.76
NFL	Feb	2011	46.1	4.9–117.1	1.13	1.95 ± 0.20		0.65
NFL	May	2011	114.1	12.0–292.4	1.5	5.44 ± 0.54		0.9
NFL	Sept	2011	53.7	4.7–231.3	1.27	3.62 ± 0.34		0.89
NFL	Integrated	–	75.4	8.2–173.0	13.2	649.9 ± 69.2	620.6 ± 36.2	0.76
MM	May	2011	12.8	2.2–26.9	2.84	24.98 ± 2.29		0.93
MM	Sept	2011	5.2	0.8–11.8	0.38	0.28 ± 0.03		0.88
MM	Integrated	–	9.1	1.6–18.1	1.74	10.0 ± 1.16	5.11 ± 0.566	0.8

Plants were grown in pots in an outdoor nursery at Palmerston North, NZ, during 2010–2011. Data are presented for endophyte mycelial biomass ELISA (MM) and *N*-formylloline ELISA (NFL), measured in leaf lamina harvested on 1 April 2010, 18 May 2010, 16 February 2011, 2 May 2011 and 29 September 2011 as well as measures (Integrated) calculated across all monthly measurements

^a^Units for NFL and MM are immunoreactive equivalent units (IRE) μg/g DW (NFL) or mg/g DW (MM)

^b^All significant at *P* < 0.01; based on square root-transformed data except for NFL May 2010, NFL integrated, MM May 2011 and MM integrated

### QTL analysis in population *I* × *S*

**Fig. 1:**
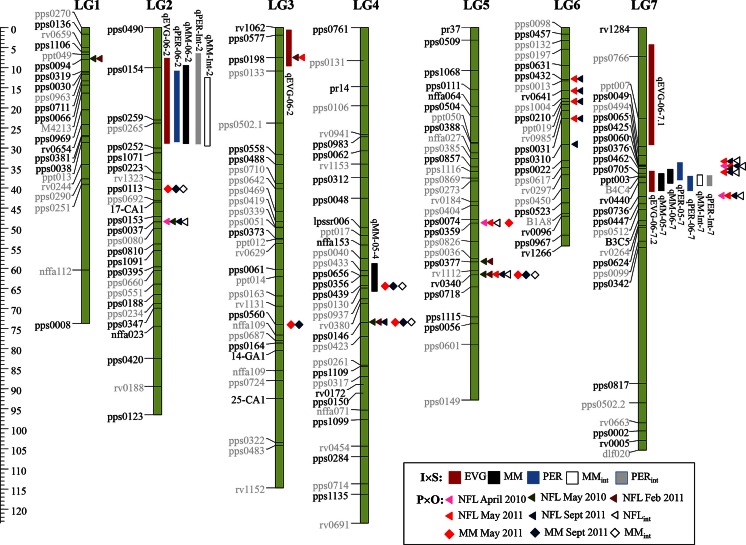
MQM QTL positions for endophyte mycelial mass (MM), peramine (PER) and ergovaline (EGV) measured in 2005, 2006 and calculated across both years (MM_in_t and PER_int_) on a genetic linkage map developed for *F*_1_ perennial ryegrass mapping population *I* × *S*. QTL 2-LOD confidence intervals are indicated by *blocks* at right of the linkage groups (QTL names as per Table 3). Locations of SSR loci (*triangle* and *diamond* *symbols*) associated by Kruskal–Wallis analysis with MM (*diamond* *symbols*) and *N*-formylloline (NFL, *triangle* *symbols*) in population *P* × *O* are also shown for individual months and calculated across months (MM_int_ and NFL_int_). Marker names are shown at *left* of linkage groups. Marker names in *black* were polymorphic in population *P* × *O*. The length of linkage groups in centimorgan (cM) is indicated by the *scale* at the *left* of the figure

A total of 11 putative QTL were identified by MQM for the traits MM, PER and EGV, at five discrete positions across four linkage groups (Fig. 1). Parental effects at most QTL were contributed from both mapping population parents ‘S’ and ‘I’ (Table 3).

**Table 3:** Detection of perennial ryegrass QTL controlling endophyte traits, by simple interval mapping (IM) and multiple QTL model mapping (MQM) in mapping population *I* × *S* in 2005, 2006 and for BLUPs calculated across both years (integrated)

LG	Trait	Year	QTL name	LOD threshold		LOD score		*V*_p_ (%)	2-LOD interval (cM)	LOD peak (cM)	Closest marker	Parental effects	
				LG wide	Genome wide	IM	MQM					*I*	*S*
2	MM	2006	qMM-06-2	2.84	3.87	4.81	6.46	15.8	9.7–29.0	28.6	pps0252	−0.42	3.62
	PER	2006	qPER-06-2	2.87	3.98	3.02	7.10	19.5	10.2–28.7	19.9	pps0265	−3.12	13.81
	EGV	2006	qEVG-06-2	2.89	3.87	5.59	5.88	15.2	7.5–29.0	23.6	pps0265	−0.48	6.49
	MM	Integrated	qMM-Int-2	2.75	3.95	4.67	5.71	14.9	12.1–29.4	28.6	pps0265	−0.25	1.75
	PER	Integrated	qPER-Int-2	2.82	3.95	3.07	4.12	12.1	6.1–29.0	19.9	pps0265	−1.46	2.96
3	EGV	2006	qEVG-06-3	2.89	3.87	2.52	3.98	6.6	0.8–9.6	6.9	pps0198	−2.28	−3.73
4	MM	2005	qMM-05-4	3.05	3.95	3.65	4.15	7.7	58.2–66.0	62.0	pps0656	1.29	0.53
7	MM	2005	qMM-05-7	3.02	3.95	3.96	5.09	9.7	36.4–40.3	38.7	rv0440	0.75	−1.54
	PER	2005	qPER-05-7	2.79	3.94	4.39	4.40	8.6	33.5–37.8	36.4	pps0705	1.61	−7.20
	MM	2006	qMM-06-7	2.74	3.87	5.14	7.01	14.2	35.2–38.6	37.3	B4C4	2.57	−2.73
	PER	2006	qPER-06-7	2.92	3.98	8.26	10.56	16.7	37.2–40.3	38.7	rv0440	8.20	−9.82
	EGV	2006	qEVG-06-7.1	2.89	3.87	7.49	5.61	10.7	4.5–29.0	19.7	ppt007	6.87	2.36
	EGV	2006	qEVG-06-7.2	–	–	–	5.88	10.6	35.4–40.8	37.3	B4C4	5.02	−1.82
	MM	Integrated	qMM-Int-7	2.91	3.95	5.82	6.82	14.1	36.7–38.9	37.3	B4C4	1.36	−1.50
	PER	Integrated	qPER-Int-7	2.94	3.95	7.91	9.41	21.0	37.0–39.9	38.7	rv0440	2.98	−3.55

Parental effects: *I* = substitution effect of alleles from maternal parent; *S* = substitution effect of alleles from paternal parent; sign indicates direction of effect; number indicates magnitude of effect. For EGV, QTL were only detected using the 2006 data set

*MM* endophyte mycelial biomass ELISA, *PER* peramine, *EGV* ergovaline, *LG* linkage group, *LOD* threshold logarithm of the odds score for declaring significant QTL at *P* ≤ 0.05, *cM* centimorgan, *V*_p_ phenotypic variation explained by MQM QTL, *2-LOD* *interval* 2-LOD score support interval for MQM QTL position

Three genomic regions influencing MM occurred on LG2, LG4 and LG7 (Table 3; Fig. 1). Only one QTL, on LG7, was detected in both 2005 and 2006, with the others significant for single years only (LG4 in 2005 and LG2 in 2006), consistent with the significant genotype-by-year interaction determined from REML analysis. The direction of parental effects at the LG7 QTL was conserved between 2005 and 2006, inferring a common genetic basis for the QTL. MM QTL together accounted for 17.4 % of V_p_ in population *I* × *S* in 2005 and 23.2 % in 2006.

Three PER QTLs were identified and all co-located with MM QTL. An LG7 QTL for PER, previously detected by interval mapping (Koulman et al. 2009), occurred in both 2005 (*V*_p_ 8.6 %) and 2006 (*V*_p_ 16.7 %). In both instances, the parental effects of the PER QTL were in alignment with the MM QTL at the same position. A third PER QTL on LG2 (*V*_p_ 19.5 %) was detected only in 2006 and together the two 2006 PER QTL accounted for total *V*_p_ of 36.2 % (Table 3). As with the LG7 position, the magnitude and direction of parental effects for the LG2 MM and LG2 PER QTL were in agreement (Table 3). The year-to-year variation in PER QTL detection was consistent with the strong genotype-by-year interaction determined from REML analysis of this trait.

A total of four EGV QTL accounted for 43.4 % *V*_p_ in population *I* × *S* in 2006 (Table 3): two of the EGV QTL coincided with the LG2 and LG7 QTL positions determined for both MM and PER (Fig. 1; Table 3), while a further two (LG3 and LG7) occurred independently of MM and PER. The LG3 QTL was subsignificant under interval mapping but progressed above the significance threshold using the MQM model (Table 3). A second LG7 QTL also only emerged under MQM, separating a single putative but significant interval mapping QTL into two, closely linked QTL with opposing parental effects. No significant QTLs were detected for EGV in 2005 due to the lack of significant genotypic variation.

QTL analysis based on BLUPs that were estimated from analysis across both years (MM_int_ and PER_int_) identified significant QTL at the LG2 and LG7 positions, for both traits (Table 3; Fig. 1). In each case, these corresponded with QTL identified in one or both of the individual years.

### Single-marker analysis in population *P* × *O*

In the *P* × *OF*_1_ progeny, 147 of the 153 SSR primer pairs tested amplified PCR products of a size consistent with previous evaluations in ryegrass (Faville et al. 2004; Sartie et al. 2011). Of these markers, 107 yielded alleles that segregated within the population at a frequency of between 0.20 and 0.80. Data from this group of 107 SSR markers were analysed by Kruskal–Wallis analysis using the NFL and MM BLUP data sets. Alleles at 17 SSR loci were significantly associated with NFL, and six loci were associated with MM (Table 4; Fig. 1). All significant alleles occurred at a frequency of 0.20–0.40 (Table 4). Significant loci for NFL occurred on all LGs, at nine genomic locations defined either as single locus positions on LG1, LG2 and LG3 or as uninterrupted blocks of significant loci on LG4 (pps0439 to pps0146), LG5 (pps0359 to rv0340), LG6 (pps0432 to pps0310) and LG7 (pps0065 to pps0736). At a given significant marker locus, the direction of parental effects was conserved amongst the different NFL measurements at that position (Table 4). Parental effects were also conserved amongst loci within the uninterrupted blocks on LG4–LG7 (Table 4). The significance of the trait-associated markers varied across the five sets of NFL measurements, but there was no clear pattern that indicated an influence of season—14 of the 17 SSR loci associated with NFL were significant for measurements that were taken in different seasons (Table 4). This suggests an interaction between genotype and environmental or management factors that are not linked to season per se.

**Table 4:** Marker alleles significantly associated by Kruskal–Wallis analysis with NFL (*N*-formylloline) and MM (endophyte mycelial biomass) BLUPs estimated in population *P* × *O* in different months during 2010–2011, as well for BLUPs integrating values across monthly sampling times (NFL_int_ and MM_int_)

LG	Position (cM)^a^	Marker_allele (bp)	Allele frequency	Trait	Date	Effect^b^	Mean (0)^c^	Mean (1)^c^	*P* value	*P* value (NFL_int_, MM_int_)
1	7.9	pps0094_134	0.20	NFL	May 2010	–	74.4	59.1	<0.01	
					Feb 2011	–	61.5	46.5	<0.005	
2	40.3	pps0113_288	0.27	MM	May 2011	+	10.6	13.4	<0.005	<0.005
					Sept 2011	+	4.1	5.4	<0.005	
2	48.4	pps0037_180	0.37	NFL	April 2010	+	90.7	118.7	<0.005	<0.005
					May 2010	+	55.7	65.7	<0.01	
					Sept 2011	+	47.9	63.9	<0.005	
3	7.5	pps0198_265	0.40	NFL	Feb 2011	–	57.0	42.2	<0.01	
					May 2011	–	128.7	113.6	<0.005	
3	74.0	pps0560_126	0.27	MM	May 2011	+	11.1	13.5	<0.005	
					Sept 2011	+	4.7	5.4	<0.01	
4	64.3	pps0439_271	0.21	MM	May 2011	–	15.5	12.1	<0.0005	<0.0005
					Sept 2011	–	6.1	4.9	<0.005	
4	73.6	pps0146_256	0.23	NFL	May 2010	–	70.5	58.7	<0.01	
					Feb 2011	–	58.6	46.7	<0.005	
					Sept 2011	–	71.5	54.3	<0.01	
				MM	May 2011	–	14.6	12.3	<0.005	<0.005
					Sept 2011	–	5.9	5.1	<0.01	
5	48.7	pps0359_270	0.33	NFL	April 2010	–	121.2	103.1	<0.005	<0.005
					May 2011	–	129.6	110.4	<0.01	
				MM	May 2011	–	14.3	12.2	<0.01	
5	58.4	pps0377_171	0.39	NFL	May 2010	–	69.6	59.6	<0.01	
					Feb 2011	–	58.6	43.4	<0.0001	
5	61.6	rv0340_136	0.38	NFL	May 2010	–	68.5	55.1	<0.0005	<0.0005
					Feb 2011	–	53.1	42.9	<0.005	
					May 2011	–	129.6	103.7	<0.0005	
					Sept 2011	–	64.1	48.1	<0.005	
				MM	May 2011	–	13.7	11.4	< 0.001	<0.005
					Sept 2011	–	5.6	4.8	<0.01	
6	12.9	pps0432_225	0.29	NFL	May 2011	+	104.9	126.2	<0.01	
					Sept 2011	+	49.3	61.6	<0.001	
6	15.7	rv0641_232	0.25	NFL	May 2011	+	102.2	125.3	<0.01	
					Sept 2011	+	48.2	61.3	<0.01	
6	18.4	pps0210_209	0.26	NFL	May 2011	+	100.4	126.4	<0.001	
					Sept 2011	+	46.3	62.1	<0.001	
6	22.7	pps0031_206	0.25	NFL	May 2011	+	101.2	125.7	<0.005	
					Sept 2011	+	44.8	62.3	<0.0005	
6	29.4	pps0310_267	0.23	NFL	Sept 2011	+	46.0	61.2	<0.005	
7	34.2	pps0065_139	0.31	NFL	May 2011	+	103.4	127.0	<0.005	<0.005
					Sept 2011	+	43.5	64.6	<0.0001	
7	34.3	pps0425_399	0.31	NFL	April 2010	+	86.2	118.6	<0.005	<0.001
					May 2011	+	102.7	127.3	<0.001	
					Sept 2011	+	45.6	63.7	<0.0005	
7	34.4	pps0060_134	0.25	NFL	Sept 2011	+	47.5	62.0	<0.005	
7	34.6	pps0376_205	0.30	NFL	May 2011	+	101.2	128.9	<0.0005	<0.005
					Sept 2011	+	43.7	64.4	<0.0001	
7	42.1	pps0736_176	0.31	NFL	April 2010	+	89.4	116.7	<0.01	<0.001
					May 2011	+	102.8	128.6	<0.001	
					Sept 2011	+	45.8	64.4	<0.0005	

*LG* linkage group

^a^Marker position estimated on *I* × *S* genetic linkage map (cM = centimorgan)

^b^‘+’ presence of the allele increases the trait value; ‘−’ presence of the allele decreases the trait value

^c^Mean phenotypic value of individuals with allele absent (data back-transformed where appropriate)

Six significant marker loci for MM, which was measured at two of the five time points (May 2011 and September 2011), were located at positions on LG2–LG5, and three of these six loci (pps0146 on LG4; pps0359 and rv0340 on LG5) were co-located with NFL loci (Fig. 1). At these common loci, the direction of parental effects was conserved between MM and NFL. All but one of the six marker loci (pps0359) were significant for both the May and September MM measures, and direction of parental effects was consistently the same for both.

Analysis using NFL_int_ and MM_int_ integrating data across all months identified a subset of the markers, from amongst those that were significant for individual measurements (Table 4; Fig. 1), which have potentially heightened stability across time or environments.

### Comparative analysis between mapping populations

Assuming conservation of marker genomic locations between the two populations, *I* × *S* and *P* × *O*, three of the significant marker positions in population *P* × *O* co-aligned with QTL intervals in population *I* × *S* (Fig. 1). This accounts for 60 % of the QTL locations detected in population *I* × *S* and 33 % of those in *P* × *O*. The locus pps0439 on LG4 was associated with MM in population *P* × *O* and occurred within the confidence interval of the MM 2005 QTL in population *I* × *S* (Fig. 1). The pps0198 locus on LG3 was significant for NFL and aligned with the *I* × *S* QTL interval for EGV 2006. Of most interest, on LG7 a group of closely spaced marker loci significant for NFL occurred at the same position as the *I* × *S* QTL cluster for PER, EGV and MM (Fig. 1).

## Discussion

Host-mediated genetic regulation of fungal endophyte traits in forage grasses is supported by evidence from various studies (Latch 1994; Adcock et al. 1997; Schmid et al. 2000; Easton et al. 2002) and has shown to be heritable (Adcock et al. 1997; Easton et al. 2002). However, little is known about the molecular basis of the plant–fungus interaction, particularly from the host plant perspective. Building on an earlier, interval mapping QTL analysis of PER in population *I* × *S* (Koulman et al. 2009), our investigation provides additional evidence for a host plant quantitative genetic influence on *inplanta* endophyte phenotypes in perennial ryegrass and, further, delineates a proportion of that control to discrete regions of the ryegrass genome, some of which are conserved across plant genetic background × endophyte strain combinations.

Analysis of data from two ryegrass mapping populations, *I* × *S* and *P* × *O*, revealed significant genotypic variation both for the concentration of alkaloids in herbage (PER and EGV in population *I* × *S*; NFL in population *P* × *O*) and for endophyte mycelial mass (MM), evidenced by genotypic variance components and clonal repeatability (*R*_c_) estimates. The estimates of genotypic variation indicate the presence of potential genetic variation for these traits in both mapping populations *I* × *S* and *P* × *O*. An exception was EGV 2005 in population *I* × *S*, for which levels were low and occupied a relatively narrow range. This restricted range is most likely influenced by both seasonal variation and the age of the herbage evaluated. EGV concentration in ryegrass herbage in autumn is typically in decline after a late summer peak (Bluett et al. 2005; Thom et al. 2012b), and EGV accumulates at low concentrations in leaf laminae, compared to leaf sheaths and stem (Watson et al. 1999; Spiering et al. 2005), but does tend towards higher levels in younger leaves of the plant (Belesky and Hill 1997; Spiering et al. 2005). The 2005 herbage samples, predominantly leaf laminae, were harvested 4 weeks post-defoliation, whereas higher EGV levels were detected in 2006 when 2-week-old regrowth (with a higher proportion of younger leaves) was evaluated. Estimation of BLUP-based adjusted means from the 2005 data set for both EGV and PER was also likely affected by design aspects of the experiment, specifically the smaller number of clonal replicates analysed and the low-resolution mass spectrometry-based metabolomic methods applied (Koulman et al. 2009). This was reflected in the *R*_c_ estimated for PER. *R*_c_ is indicative of an upper limit for heritability but is not a fixed characteristic of the trait, being affected also by features of the evaluation including measurement precision. *R*_c_ for PER was low in 2005 but increased considerably in 2006 when all three clonal replicates were utilised in the analysis and plant samples were assayed by targeted DIMS(MS) as opposed to the potentially less precise untargeted DIMSMS methodology applied in 2005. The disparity in approaches between years may also have influenced the strong contribution of the genotype-by-year variance component observed for PER, relative to the genotypic variance component. Notwithstanding this, in both populations the ratios of genotypic to genotype-by-sampling time variance components indicate a stronger influence of sampling time on alkaloids (PER and NFL) compared with MM.

NFL levels in population *P* × *O* (4.7–313.4 immunoreactive equivalents μg/g DW) were consistent with previous measurement from *E*. sp. FaTG-3 endophytes within a ryegrass host, from an outdoor pot study reported by Easton et al. (2009) (30–400 μg NFL/g DW by gas chromatography). Seasonal variation in NFL was partly consistent with the seasonal pattern reported for loline-producing endophyte strains in their native plant hosts in field conditions. In meadow fescue under New Zealand conditions, leaf lolines peak in late spring and then again in late summer, before declining through autumn (Patchett et al. 2011). A similar late summer spike in lolines was described for endophyte-infected tall fescue in North America and was associated with low water availability and dry matter accumulation (Bush and Fannin 2009). In our data set, late summer NFL (February 2011) was relatively low when compared to measurements at other times of the year. This may be a consequence of the novel nature of the host–endophyte association in population *P* × *O*, but also the growth environment was atypical of summer field conditions, as the plants were grown in pots that were irrigated daily and had been repotted into fresh media only 2 months prior to the February leaf harvest. Therefore, although both experiments were conducted outdoors and exposed to predominantly natural climatic influences, care must be taken in comparing these studies with field studies.

The central finding of this investigation was the identification, within two genetically distinct mapping populations, of at least 11 regions of the host plant genome affecting either alkaloid concentration or MM or both. Earlier research, in an independent perennial ryegrass mapping population, reported host QTL that influence MM, measured by ELISA (van Zijll de Jong et al. 2005), but the genomic location of these regions were not provided to allow comparison with the current findings.

Co-localisation and conservation of parental effects at QTL for EGV, PER and MM on LG2 and LG7 of the *I* × *S* map confirm that the host plant imparts genetic regulation of endophyte mycelial mass levels in shoot tissues and, further, implies that the effect on alkaloid expression mediated by these two QTL is influenced by the quantity of endophyte mycelia present in the leaf. Nevertheless, the limited resolution rendered by QTL mapping means the possibility of closely linked causative genes, acting independently on endophyte alkaloid levels and endophyte biomass, cannot be fully discounted.

Conversely, alkaloid QTL detected at genomic locations in the absence of MM QTL suggest host factors that directly affect metabolite expression by the endophyte, rather than by modulation of endophyte mycelial mass levels. In population *I* × *S*, MM-independent QTL for EGV were identified on LG3 and LG7, in contrast to PER for which all QTL consistently co-located with MM QTL. This indicates that host genetic influence on EGV expression may not be exclusively a consequence of the quantity of endophyte mycelial mass in leaf tissues. The contrasting relationships of PER and EGV with endophyte biomass were also reflected in the 2006 phenotypic correlations of MM with EGV (*r*^2^ = 0.06) and PER (*r*^2^ = 0.41). These results are consistent with Spiering et al. (2005), who reported endophyte biomass in ryegrass tillers accounted for 20 and 31 % of the variation in EGV and PER, respectively, and Easton et al. (2002) who determined that 65 % of the genetically controlled variation in PER was accounted for by endophyte mycelial mass, while the equivalent proportion for EGV was 41 %. The comparatively weak MM–EGV correlation in our data set may be due to the tissue type being predominantly leaf laminae, in contrast to pseudostem (Easton et al. 2002) and whole tiller (Spiering et al. 2005) samples of other studies which potentially assayed a larger proportion of the predominantly basally distributed EGV (Spiering et al. 2005). The identification of independent QTL for EGV and for PER–MM supports earlier conclusions (Roylance et al. 1994; Easton et al. 2002) that it should be possible to select within host breeding populations for reduced levels of the livestock toxin EGV in biomass, without eliciting a significant, concurrent decline in the insect-deterrent PER.

Evaluation of the NFL–MM relationship in population *P* × *O* was limited to only two of the five measurement time points, but those data imply a condition comparable to that of EGV, with which NFL shares a similarly basal tissue distribution in the tiller (Justus et al. 1997). The phenotypic correlation between NFL and MM in leaf laminae was significant but weak (*r*^2^ = 0.17), and markers associated with NFL-only occurred at positions on LG3, LG6 and LG7, indicative of a direct host genome influence on NFL production. The lack of significant marker effects for MM in this LG7 region is notable because it coincides with an *I* × *S* QTL confidence interval that influences both alkaloids and MM. This disparity may be an outcome of the effects of different genes or alleles within this chromosomal region or reflect differences in the distribution of endophyte in the shoot tissues of the respective populations.

The stability of QTL, which may subsequently become candidates for marker-assisted selection or targets for the elucidation of functional gene variants (Price 2006; Barrett et al. 2008), is a key factor for breeding purposes. Variation in QTL occurrence was seen in both populations across different measurement time points, suggesting that intrinsic plant age, physical environmental or management factors interact with the host genes affecting endophyte biomass and alkaloid accumulation. Even so, stability was observed for a number of QTL affecting endophyte alkaloid accumulation or endophyte biomass. Within *I* × *S* and *P* × *O*, there were QTL that recurred at different sampling times, and additionally, a subset of these associations were confirmed when using multisampling BLUPs. Furthermore, there were genomic regions that were significant in both mapping populations, despite the populations being genetically distinct from one another and infected with different endophyte species (one a naturalised association and one a novel association). Cross-population QTL positions on LG3 (EGV and NFL), LG4 (MM) and, in particular, LG7 (MM, PER, EGV, NFL) point to the presence of host gene effects that are conserved across genetic backgrounds and which might be leveraged to improve endophyte trait expression in a broad range of host population/species × endophyte combinations. Fixation of favourable alleles at these loci in a population, be it via MAS or conventional recurrent selection, may serve to attenuate the genetic incompatibility associated with novel host–endophyte associations (Saikkonen et al. 2010). Additionally, from a practical perspective this suggests that, where compatibility has been achieved for a particular novel host–endophyte association within a given population, a degree of endophyte cross-compatibility might be established, such that future host–endophyte associations in that population, using a new endophyte variant, may achieve stability more rapidly and therefore cost-effective.

Elucidation of genes underlying the host plant–endophyte interaction cannot be based on a low-resolution QTL study alone. However, having a robust estimate of QTL locations may support candidate gene identification when considered in conjunction with information from, for example, transcriptomic (Eaton et al. 2010; Zhang et al. 2011) or metabolomic datastreams (Cao et al. 2008). Weak induction of the host plant defence response has been identified as a factor in the successful promotion of functional plant–fungal symbioses (García-Garrido and Ocampo 2002), and this is potentially the case also for the perennial ryegrass–*E*. *festucae* subsp. *lolii* association (Zhang et al. 2011). In that framework, our results point towards a potential role for host defence-related (DR) genes as candidate genes influencing the regulation of endophyte-related traits *inplanta*. Firstly, the LG7 QTL region repeatedly highlighted in the current study is co-linear with the wheat homologous group 7 chromosomes (Jones et al. 2002; Sim et al. 2005) characterised by a relatively high density of DR genes (Li et al. 1999), and a number of DR gene sequences have also been mapped to ryegrass LG7 (Faville et al. 2004). Secondly, Zhang et al. (2011) found a positive correlation in ryegrass between the presence of endophyte and levels of the pathogenesis-related protein PR-10, inferring a role for this host-derived protein in a functional symbiosis. The ryegrass PR-10 nucleotide sequence (HQ229927) matches to a rice genome position at 10.6 Mb on chromosome 3 (BLASTn e-value 3.6e^−42^) that is contiguous with the LG4 MM QTL region identified in the current study for both mapping populations (pps0040 9.2 Mb, 3.0e^−78^ to pps0130 13.3 Mb, 1.0e^−56^). This suggests that the PR-10 locus might be a candidate for the QTL detected here.

Endophyte biomass can be estimated using quantitative PCR (qPCR) (Panaccione et al. 2001; Young et al. 2005; Rasmussen et al. 2007) although in this study it was determined by ELISA. Antibodies used in ELISAs have been raised against the soluble antigen fraction from *E*. *festucae* var. *lolii* endophyte cultures (Musgrave and Fletcher 1986; Ball et al. 1995). Biochemical characterisation of these antigens suggests that the major antigen is a polysaccharide moiety of a protein–lipopolysaccharide complex (Musgrave and Fletcher 1986). A new assay was developed and validation studies undertaken for the current research. Improved assay performance was achieved by replacing the sandwich format described by Ball et al. (1995) with a competitive ELISA characterised by increased assay sensitivity with reduced sample matrix interferences. The herbage extraction procedure used was similar to that of Easton et al. (2002) except that extraction was with PBST at 30 °C, and this was continued for 3 h rather than 30 min.

In conclusion, these results are further evidence for a host plant quantitative genetic influence on endophyte trait phenotypes in ryegrass. QTL analysis of the genetic architecture of the host plant–endophyte interaction has ascribed a proportion of that control to discrete regions of the ryegrass genome. The MM ELISA developed for the current study provides a cost-effective and high-throughput method for determining endophyte mycelial mass and assay sensitivity is such that the method is suitable for the analysis of plant material. The methodology has been applied extensively here and is a useful research tool where analysis of a large number of samples is required or when sample size is limited. Host genetic regulation of endophyte alkaloid levels via the discovered QTL either acts directly or is mediated by the quantity of endophyte present in the leaf tissue. A subset of QTL, on LG3, LG4 and LG7, are conserved across plant genetic background × endophyte variant combinations and represent strong candidates for further development, for MAS for endophyte traits in grass breeding programmes and for future investigation of candidate plant genes underlying the host–endophyte interaction. The expression of lolines in ryegrass, by developing novel symbioses with fescue-derived, loline-producing endophyte variants, provides a mechanism for improving plant resistance to a broad range of insect herbivores, but expression levels are low compared with those in the host fescue (Easton et al. 2009). The identification of QTL associated with NFL may provide a marker-assisted breeding basis for genetic improvement of loline expression in ryegrass hosts. Variation in QTL occurrence was a key finding of this study, meaning that further research is needed to validate the current marker–trait associations for different endophyte variants and to determine their stability to variation in the environment.

## Electronic supplementary material

Fig. S1 Frequency distributions of BLUP adjusted means for traits endophyte mycelial mass (MM), peramine (PER) and ergovaline (EGV), measured in 2005, 2006 and calculated over both years (Integrated), in perennial ryegrass mapping population *I* × *S* *F*_1_ progeny (n = 200 plants). NIU = normalised intensity units; IRE = immunoreactive equivalents. Arrows indicate trait means for parent ‘I’. (TIFF 15848 kb)

Fig. S2 Frequency distributions of BLUP adjusted means in perennial ryegrass population *P* × *O* *F*_1_ progeny (n = 285 plants), for traits (a) *N*-formylloline (NFL) measured in April 2010, May 2010, February 2011, May 2011, September 2011 and calculated across all months (Integrated) and (b) endophyte mycelial mass (MM) measured in May 2011, September 2011 and calculated over both months (Integrated). IRE = immunoreactive equivalents. Data for all traits are square root-transformed, except MM May 2011, MM Integrated, NFL May 2010 and NFL Integrated (all non-transformed). (TIFF 8618 kb)

Supplementary material 3 (DOCX 40 kb)

## Abbreviations

DIMSMS: Direct infusion tandem mass spectrometry
EGV: Ergovaline
MM: Endophyte mycelial mass
m/z: Mass-to-charge ratio
NFL: *N*-formylloline
PER: Peramine
*R*_c_: Clonal repeatability

## References

[CR1] Adcock RA, Hill NS, Bouton JH, Boerma HR, Ware GO (1997). Symbiont regulation and reducing ergot alkaloid concentration by breeding endophyte-infected tall fescue. J Chem Ecol.

[CR2] Ball OJ, Prestidge RA, Sprosen JM (1995). Interrelationships between *Acremonium**lolii*, peramine, and lolitrem B in perennial ryegrass. Appl Environ Microbiol.

[CR3] Ball OJ-P, Miles CO, Prestidge RA (1997). Ergopeptine alkaloids and *Neotyphodium**lolii*-mediated resistance in perennial ryegrass against adult *Heteronychus**arator* (Coleoptera: Scarabaeidae). J Econ Entomol.

[CR4] Ball OJP, Coudron TA, Tapper BA, Davies E, Trently D, Bush LP, Gwinn KD, Popay AJ (2006). Importance of host plant species, *Neotyphodium* endophyte isolate, and alkaloids on feeding by *Spodoptera**frugiperda* (Lepidoptera: Noctuidae) larvae. J Econ Entomol.

[CR5] Barker SJ, Duplessis S, Tagu D (2002). The application of genetic approaches for investigations of mycorrhizal symbioses. Plant Soil.

[CR6] Barrett B, Baird I, Woodfield D, Yamada T, Spangenberg G (2008). White clover seed yield: a case study in marker assisted selection. Molecular breeding of forage and turf: proceedings of the 5th international symposium, Sapporo, Japan.

[CR7] Belesky DP, Hill NS (1997). Defoliation and leaf age influence on ergot alkaloids in tall fescue. Ann Bot.

[CR8] Bluett SJ, Thom ER, Clark DA, Macdonald KA, Minneé EMK (2005). Effects of perennial ryegrass infected with either AR1 or wild endophyte on dairy production in the Waikato. N Z J Agric Res.

[CR9] Brooks TD, Williams WP, Windham GL, Willcox MC, Abbas HK (2005). Quantitative trait loci contributing resistance to aflatoxin accumulation in the maize inbred Mp313E. Crop Sci.

[CR10] Bush L, Fannin FF, Fribourg HA, Hannaway DB, West CP (2009). Alkaloids. Tall fescue for the twenty-first century.

[CR11] Cao M, Koulman A, Johnson LJ, Lane GA, Rasmussen S (2008). Advanced data-mining strategies for the analysis of direct-infusion ion trap mass spectrometry data from the association of perennial ryegrass with its endophytic fungus, *Neotyphodium**lolii*. Plant Physiol.

[CR12] Churchill DA, Doerge RW (1994). Empirical threshold values for quantitative trait mapping. Genetics.

[CR13] Easton HS (2007). Grasses and *Neotyphodium* endophytes: co-adaptation and adaptive breeding. Euphytica.

[CR14] Easton HS, Latch GCM, Tapper BA, Ball OJ-P (2002). Ryegrass host genetic control of concentrations of endophyte-derived alkaloids. Crop Sci.

[CR15] Easton HS, Lyons TB, Mace WJ, Simpson WR, Bonth ACMd, Cooper BM, Panckhurst KA (2009). Differential expression of loline alkaloids in perennial ryegrass infected with endophyte isolated from tall fescue. Grassl Res Pract Ser.

[CR16] Eaton CJ, Cox MP, Ambrose B, Becker M, Hesse U, Schardl CL, Scott B (2010). Disruption of signaling in a fungal-grass symbiosis leads to pathogenesis. Plant Physiol.

[CR17] Falconer DS (1989). Introduction to quantitative genetics.

[CR18] Faville M, Vecchies A, Schreiber M, Drayton M, Hughes L, Jones E, Guthridge K, Smith K, Sawbridge T, Spangenberg G, Bryan G, Forster J (2004). Functionally associated molecular genetic marker map construction in perennial ryegrass (*Lolium**perenne* L.). Theor Appl Genet.

[CR19] Faville MJ, Jahufer MZZ, Hume DE, Cooper BM, Pennell CGL, Ryan DL, Easton HS (2012). Quantitative trait locus mapping of genomic regions controlling herbage yield in perennial ryegrass. N Z J Agric Res.

[CR20] Fletcher LR, Easton HS, Bacon C, Hill N (1997). The evaluation and use of endophytes for pasture improvement. Neotyphodium/grass interactions.

[CR21] Galván G, Kuyper T, Burger K, Keizer LCP, Hoekstra R, Kik C, Scholten O (2011). Genetic analysis of the interaction between *Allium* species and arbuscular mycorrhizal fungi. Theor Appl Genet.

[CR22] García-Garrido JM, Ocampo JA (2002). Regulation of the plant defence response in arbuscular mycorrhizal symbiosis. J Exp Bot.

[CR23] GenStat (2006) GenStat for Windows. Release 9.1. vol 9th edn, 9.1 edn. VSN International Ltd., Oxford

[CR24] GenStat (2012) GenStat for Windows. Release 15.2. vol 15th edn. VSN International Ltd., Oxford

[CR25] Hill NS, Bouton JH, Thompson FN, Hawkins L, Hoveland CS, McCann MA (2002). Performance of tall fescue germplasms bred for high- and low-ergot alkaloids. Crop Sci.

[CR26] Horsley R, Schmierer D, Maier C, Kudrna D, Urrea C, Steffenson B, Schwarz P, Franckowiak J, Green M, Zhang B (2006). Identification of QTLs associated with *Fusarium* head blight resistance in barley accession CIho 4196. Crop Sci.

[CR27] Johnson L, Bonth AM, Briggs L, Caradus J, Finch S, Fleetwood D, Fletcher L, Hume D, Johnson R, Popay A, Tapper B, Simpson W, Voisey C, Card S (2013). The exploitation of epichloae endophytes for agricultural benefit. Fungal Divers.

[CR28] Jones ES, Mahoney NL, Hayward MD, Armstead IP, Jones JG, Humphreys MO, King IP, Kishida T, Yamada T, Balfourier F, Charmet G, Forster JW (2002). An enhanced molecular marker based genetic map of perennial ryegrass (*Lolium**perenne*) reveals comparative relationships with other Poaceae genomes. Genome.

[CR29] Jung GA, van Wijk AJP, Hunt WF, Watson CE (1996) Ryegrasses. In: Moser LE, Buxton DR, Casler MD (eds) Cool-season forage grasses, vol 34. ASA, CSSA and SSSA, Madison, pp 605–641

[CR30] Justus M, Witte L, Hartmann T (1997). Levels and tissue distribution of loline alkaloids in endophyte-infected *Festuca**pratensis*. Phytochemistry.

[CR31] Khaembah EN, Irving LJ, Thom ER, Faville MJ, Easton HS, Matthew C (2013). Leaf rubisco turnover in a perennial ryegrass (*Lolium**perenne* L.) mapping population: genetic variation, identification of associated QTL, and correlation with plant morphology and yield. J Exp Bot.

[CR32] Knott SA, Neale DB, Sewell MM, Haley CS (1997). Multiple marker mapping of quantitative trait loci in an outbred pedigree of loblolly pine. Theor Appl Genet.

[CR33] Koulman A, Tapper BA, Fraser K, Cao M, Lane GA, Rasmussen S (2007). High-throughput direct-infusion ion trap mass spectrometry: a new method for metabolomics. Rapid Commun Mass Spectrom.

[CR34] Koulman A, Cao M, Faville M, Lane G, Mace W, Rasmussen S (2009). Semi-quantitative and structural metabolic phenotyping by direct infusion ion trap mass spectrometry and its application in genetical metabolomics. Rapid Commun Mass Spectrom.

[CR35] Latch GCM (1994). Influence of Acremonium endophytes on perennial grass improvement. N Z J Agric Res.

[CR36] Leuchtmann A, Bacon CW, Schardl CL, White JF, Tadych M (2014). Nomenclatural realignment of *Neotyphodium* species with genus *Epichloë*. Mycologia.

[CR37] Li WL, Faris JD, Chittoor JM, Leach JE, Hulbert SH, Liu DJ, Chen PD, Gill BS (1999). Genomic mapping of defense response genes in wheat. Theor Appl Genet.

[CR38] Musgrave DR, Fletcher LR (1986). Optimisation and characterisation of enzyme-linked immunosorbent assay (ELISA) for the detection of the Acremonium loliae endophyte in *Lolium**perenne*. N Z J Agric Res.

[CR39] Nodari RO, Tsai SM, Guzmán P, Gilbertson RL, Gepts P (1993). Toward an integrated linkage map of common bean. III. Mapping genetic factors controlling host-bacteria interactions. Genetics.

[CR40] Panaccione DG, Johnson RD, Wang J, Young CA, Damrongkool P, Scott B, Schardl CL (2001). Elimination of ergovaline from a grass—*Neotyphodium* endophyte symbiosis by genetic modification of the endophyte. Proc Natl Acad Sci USA.

[CR41] Patchett B, Gooneratne R, Fletcher L, Chapman B (2011). Seasonal changes in leaf and stem loline alkaloids in meadow fescue. Crop Pasture Sci.

[CR42] Paul C, Naidoo G, Forbes A, Mikkilineni V, White D, Rocheford T (2003). Quantitative trait loci for low aflatoxin production in two related maize populations. Theor Appl Genet.

[CR43] Popay AJ, Bonos SA (2005). Biotic responses in endophytic grasses. Neotyphodium in cool-season grasses.

[CR44] Prestidge RA, Ball OJ-P, Gange AC, Brown VK (1995). A catch 22: the utilization of endophytic fungi for pest management. Multitrophic interactions in terrestrial ecosystems.

[CR45] Price AH (2006). Believe it or not, QTLs are accurate!. Trends Plant Sci.

[CR46] Ramaekers L, Galeano C, Garzón N, Vanderleyden J, Blair M (2013). Identifying quantitative trait loci for symbiotic nitrogen fixation capacity and related traits in common bean. Mol Breed.

[CR47] Rasmussen S, Parsons AJ, Bassett S, Christensen MJ, Hume DE, Johnson LJ, Johnson RD, Simpson WR, Stacke C, Voisey CR, Xue H, Newman JA (2007). High nitrogen supply and carbohydrate content reduce fungal endophyte and alkaloid concentration in *Lolium**perenne*. New Phytol.

[CR48] Rowan DD, Gaynor DL (1986). Isolation of feeding deterrents against Argentine stem weevil from ryegrass infected with the endophyte *Acremonium**loliae*. J Chem Ecol.

[CR49] Roylance JT, Hill NS, Agee CS (1994). Ergovaline and peramine production in endophyte-infected tall fescue: independent regulation and effects of plant and endophyte genotype. J Chem Ecol.

[CR50] Saikkonen K, Wäli PR, Helander M (2010). Genetic compatibility determines endophyte–grass combinations. PLoS ONE.

[CR51] Sartie A, Matthew C, Easton H, Faville M (2011). Phenotypic and QTL analyses of herbage production-related traits in perennial ryegrass (*Lolium**perenne* L.). Euphytica.

[CR52] Schardl CL, Grossman RB, Nagabhyru P, Faulkner JR, Mallik UP (2007). Loline alkaloids: currencies of mutualism. Phytochemistry.

[CR53] Schmid J, Spiering MJ, Christensen MJ, Bacon CW, White JF (2000). Metabolic activity, distribution and propagation of grass endophytes *in**planta*. Investigations using the GUS system. Microbial endophytes.

[CR54] Sewell MM, Bassoni DL, Megraw RA, Wheeler NC, Neale DB (2000). Identification of QTLs influencing wood property traits in loblolly pine (*Pinus**taeda* L.). I. Physical wood properties. Theor Appl Genet.

[CR55] Sim S, Chang T, Curley J, Warnke SE, Barker RE, Jung G (2005). Chromosomal rearrangements differentiating the ryegrass genome from the Triticeae, oat, and rice genomes using common heterologous RFLP probes. Theor Appl Genet.

[CR56] Simpson W, Schmid J, Singh J, Faville M, Johnson R (2012). A morphological change in the fungal symbiont *Neotyphodium**lolii* induces dwarfing in its host plant *Lolium**perenne*. Fungal Biol.

[CR57] Souza AA, Boscariol RL, Moon DH, Camargo LEA, Tsai SM (2000). Effects of *Phaseolus**vulgaris* QTL in controlling host-bacteria interactions under two levels of nitrogen fertilization. Genet Mol Biol.

[CR58] Spiering MJ, Lane GA, Christensen MJ, Schmid J (2005). Distribution of the fungal endophyte *Neotyphodium**lolii* is not a major determinant of the distribution of fungal alkaloids in *Lolium**perenne* plants. Phytochemistry.

[CR59] Thom ER, Popay AJ, Hume DE, Fletcher LR (2012). Evaluating the performance of endophytes in farm systems to improve farmer outcomes—a review. Crop Pasture Sci.

[CR60] Thom ER, Waugh CD, Minneé EMK, Waghorn GC (2012). Effects of novel and wild-type endophytes in perennial ryegrass on cow health and production. N Z Vet J.

[CR61] Van Ooijen JW, Boer MP, Jansen RC, Maliepaard C (2002) MapQTL 4.0^®^, software for the calculation of QTL positions on genetic maps. Plant Res Int, Wageningen, The Netherlands

[CR62] van Zijll de Jong E, Smith KF, Spangenberg GC, Forster JW, Roberts CA, West CP, Spiers DE (2005). Molecular genetic marker-based analysis of the grass-endophyte symbiosis. *Neotyphodium* in cool-season grasses.

[CR63] van Zijll de Jong E, Dobrowolski MP, Bannan NR, Stewart AV, Smith KF, Spangenberg GC, Forster JW (2008). Global genetic diversity of the perennial ryegrass fungal endophyte *Neotyphodium**lolii*. Crop Sci.

[CR64] Watson RH, Keogh RG, McDonald MF (1999) Ewe reproductive performance on growth rate of suckling-lambs on endophyte-infected perennial ryegrass pasture. In: Ryegrass endophyte: an essential New Zealand symbiosis. Grassland Research and Practice Series No. 7, vol 7. New Zealand Grassland Association, pp 19–25

[CR65] Young CA, Bryant MK, Christensen MJ, Tapper BA, Bryan GT, Scott B (2005). Molecular cloning and genetic analysis of a symbiosis-expressed gene cluster for lolitrem biosynthesis from a mutualistic endophyte of perennial ryegrass. Mol Genet Genomics.

[CR66] Young CA, Hume DE, McCulley RL (2013). Forages and pastures symposium: fungal endophytes of tall fescue and perennial ryegrass: pasture friend or foe?. J Anim Sci.

[CR67] Zhang N, Zhang S, Borchert S, Richardson K, Schmid J (2011). High levels of a fungal superoxide dismutase and increased concentration of a PR-10 plant protein in associations between the endophytic fungus *Neotyphodium**lolii* and ryegrass. Mol Plant-Microbe Interact.

