# Supplementary figures and images for: A QTL analysis of host plant effects on fungal endophyte biomass and alkaloid expression in perennial ryegrass
Source: Mol Breed. 2015 Jul 18;35(8):161. doi: 10.1007/s11032-015-0350-1 (PMC4506467; doi:10.1007/s11032-015-0350-1)

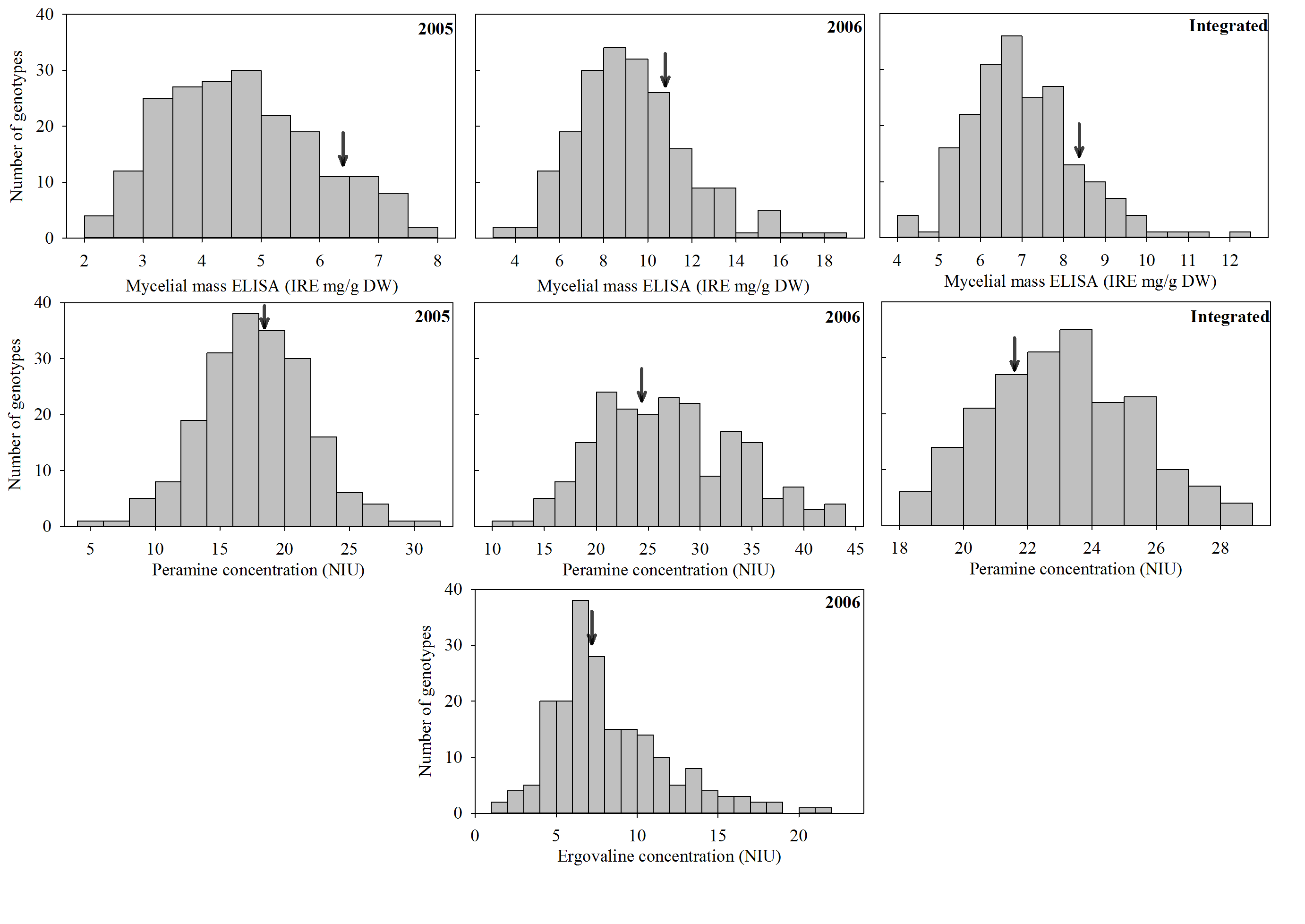

Supplement: Supplementary file 1 — Frequency distributions of BLUP adjusted means for traits endophyte mycelial mass (MM), peramine (PER) and ergovaline (EGV), measured in 2005, 2006 and calculated over both years (Integrated), in perennial ryegrass mapping population I × S F 1 progeny (n = 200 plants). NIU = normalised intensity units; IRE = immunoreactive equivalents. Arrows indicate trait means for parent ‘I’. (TIFF 15848 kb) [file 11032_2015_350_MOESM1_ESM.tif]

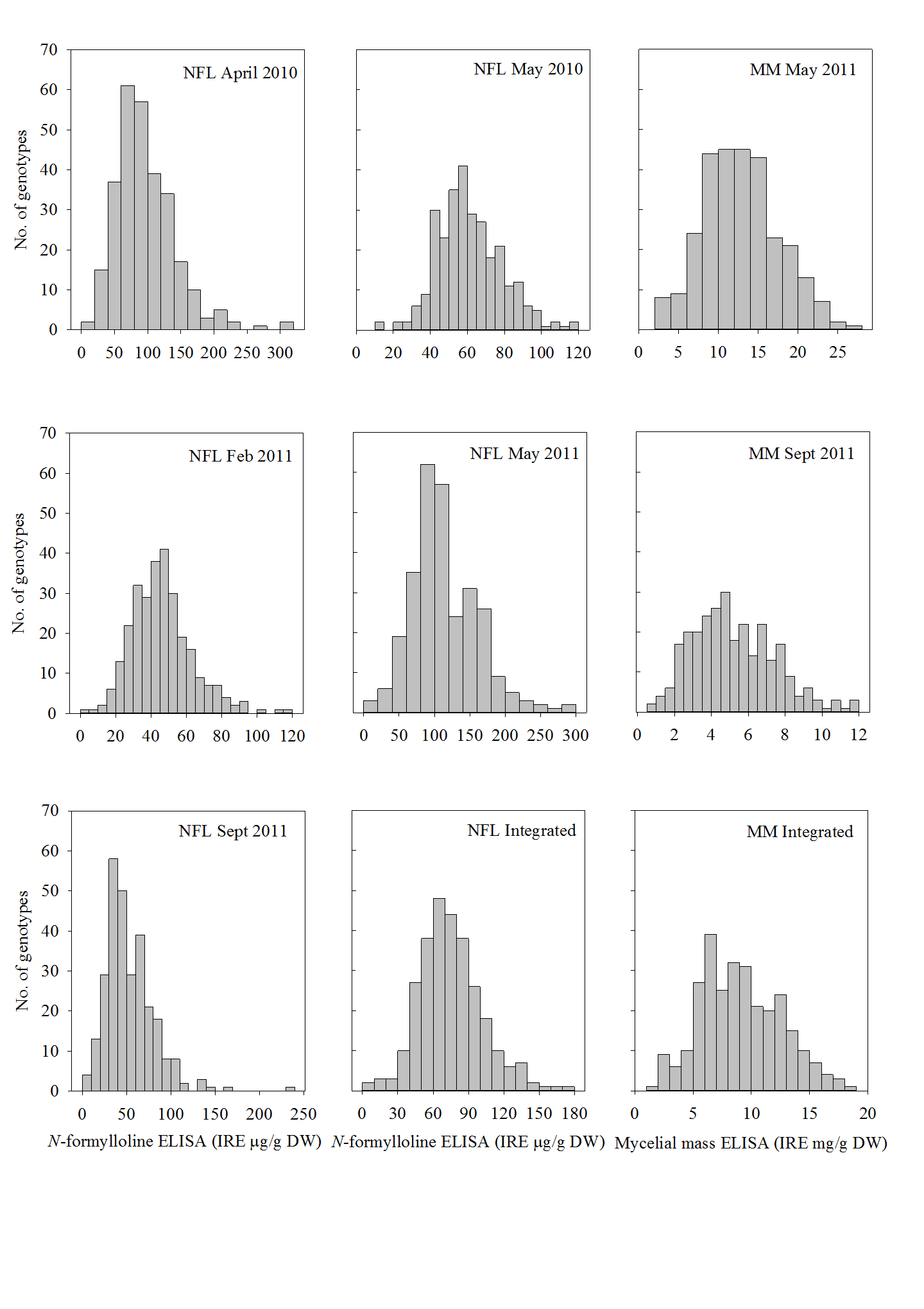

Supplement: Supplementary file 2 — Frequency distributions of BLUP adjusted means in perennial ryegrass population P × O F 1 progeny (n = 285 plants), for traits (a) N-formylloline (NFL) measured in April 2010, May 2010, February 2011, May 2011, September 2011 and calculated across all months (Integrated) and (b) endophyte mycelial mass (MM) measured in May 2011, September 2011 and calculated over both months (Integrated). IRE = immunoreactive equivalents. Data for all traits are square root-transformed, except MM May 2011, MM Integrated, NFL May 2010 and NFL Integrated (all non-transformed). (TIFF 8618 kb) [file 11032_2015_350_MOESM2_ESM.tif]
