# Supplementary material for: A QTL analysis of host plant effects on fungal endophyte biomass and alkaloid expression in perennial ryegrass
Source: Mol Breed. 2015 Jul 18;35(8):161. doi: 10.1007/s11032-015-0350-1 (PMC4506467; doi:10.1007/s11032-015-0350-1)
Supplement: Supplementary file 3 — Supplementary material 3 (DOCX 40 kb) [file 11032_2015_350_MOESM3_ESM.docx]

**Table S1.** Statistically significant phenotypic coefficients of correlation between endophyte traits assessed in: 2005 and 2006 in mapping population I×S F_1_ progeny (*P* < 0.01 if *r* ≥0.23; *P* < 0.001 if *r* >0.23); 2010 – 2011 in mapping population P×O F_1_ progeny (all significant at *P* < 0.001). EGV = ergovaline, PER = peramine, NFL = *N*-formylloline, MM = endophyte mycelial biomass.

|  | EGV 2005 | EGV 2006 | MM 2005 | MM 2006 | PER 2005 | NFL April 2010 | NFL May 2010 | NFL Feb 2011 | NFL May 2011 | NFL Sept 2011 | MM May 2011 |
| --- | --- | --- | --- | --- | --- | --- | --- | --- | --- | --- | --- |
| EGV 2006 | ns |  |  |  |  |  |  |  |  |  |  |
| MM 2005 | ns | ns |  |  |  |  |  |  |  |  |  |
| MM 2006 | ns | 0.25 | 0.46 |  |  |  |  |  |  |  |  |
| PER 2005 | 0.39 | ns | 0.22 | 0.20 |  |  |  |  |  |  |  |
| PER 2006 | ns | 0.39 | 0.34 | 0.64 | 0.23 |  |  |  |  |  |  |
| NFL May 2010 |  |  |  |  |  | 0.47 |  |  |  |  |  |
| NFL Feb 2011 |  |  |  |  |  | 0.47 | 0.54 |  |  |  |  |
| NFL May 2011 |  |  |  |  |  | 0.58 | 0.57 | 0.62 |  |  |  |
| NFL Sept 2011 |  |  |  |  |  | 0.63 | 0.51 | 0.48 | 0.68 |  |  |
| MM May 2011 |  |  |  |  |  | 0.25 | 0.22 | 0.45 | 0.35 | 0.30 |  |
| MM Sept 2011 |  |  |  |  |  | 0.31 | 0.25 | 0.40 | 0.38 | 0.47 | 0.77 |
